# Molecular Mechanisms Related to Burns, Burn Wound Healing and Scarring

**DOI:** 10.3390/ijms24108785

**Published:** 2023-05-15

**Authors:** Lars-Peter Kamolz, Andrzej Hecker

**Affiliations:** 1 Division of Plastic, Aesthetic and Reconstructive Surgery, Department of Surgery, Medical University of Graz, 8036 Graz, Austria; lars.kamolz@medunigraz.at; 2 COREMED-Centre for Regenerative Medicine and Precision Medicine, Joanneum Research Forschungsgesellschaft mbH, 8010 Graz, Austria

The process of burn injury is multifaceted and involves a whole spectrum of inflammatory responses that can have significant implications for burn patients, including local, regional, and systemic effects. Despite improvements in acute burn care, up to 70% of patients develop hypertrophic scars, which can result in functional and psychosocial consequences [1]. The current approaches to minimize scarring often fail, emphasizing the need for further research to develop effective preventive and therapeutic strategies. As medical knowledge continues to expand rapidly in all medical disciplines, keeping pace with developments in the field of burns is challenging. To address this, we curated a Special Issue titled “Molecular Mechanisms Related to Burns, Burn Wound Healing and Scarring” in the *International Journal of Molecular Sciences* to summarize recent advancements, trending topics, and the latest research in burn wound pathophysiology, healing, and scarring. This issue includes a total of seven contributions: two original articles and seven reviews providing new information about burn wound pathophysiology, burn wound healing, and scarring.

Infections and antibiotic resistance present common challenges in the treatment of burns. Souto et al. [2] discussed the latest developments in the use of nanoparticles for managing burn wound infections, regenerative medicine, and tissue engineering. The application of nanotechnology can address these challenges by incorporating antibiotics in nanoparticles, enabling targeted and localized treatment. Moreover, nanoparticles can act as drug carriers possessing therapeutic properties. Various types of nanoparticles, such as nanoemulsions, polymeric nanoparticles, metal nanoparticles, and nanogels, have been proposed to enhance antimicrobial activity [2]. Future perspectives include the use of progenitor cells and mesenchymal stem cells, combined with nanoparticles, for a synergistic effect in treating burns [2]. Stem cell-based therapies have been shown to improve the rate and quality of wound healing in younger patients [3]. However, elderly burn patients have poorer outcomes than younger patients, and the potential use of stem cells in this population remains unexplored. In a review, Elloso et al. [3] highlighted the possible use of mesenchymal stem cells in burns in the elderly population. These cells can accelerate healing in superficial partial thickness burns and donor site wounds, improve graft take and prevent graft breakdown, stimulate growth factor secretion and cell proliferation, modulate impaired immune responses, and enhance vascularization. The broad potential of mesenchymal stem cells suggests various avenues for improving outcomes in different aspects of burns, especially in the elderly population.

Hypertrophic scar development is a common problem after burn injuries, with reported prevalence rates ranging from 32% to 72% [4]. Prolonged inflammation and delayed wound healing contribute to scar formation. The process of burn wound healing consists of three phases: inflammatory, proliferative, and maturation. Based on the healing phases, Čoma et al. [5] described the interactions between different types of cells, including fibroblasts, immune cells, and epithelial cells, leading to hypertrophic scarring. Therefore, a comprehensive understanding of the molecular mechanisms underlying each phase seems crucial for developing effective therapy strategies for nearly scarless healing. Excessive scar formation after a burn is often associated with a reduced quality of life, and various products and physical therapies are available for its treatment. However, clinical efficacy remains suboptimal, and managing hypertrophic scarring continues to be challenging. Koller [6] outlined the mechanosensitive aspects of cell biology and the possible role of different wound-healing phases in these aspects. An appropriate mechanical stimulus can positively impact scar tissue by causing functional alignment through mechanotransduction, leading to positive outcomes in scar tissue. Connective tissue resistance increases in the proliferation and remodulation phase, indicating that an “overdose” of manual scar therapy in these phases may be more effective. Although manual scar therapy has demonstrated an improvement in scar region mobility, the current state of mechanotransduction research does not permit direct translation into clinical treatment [6].

In addition to hypertrophic scars, post-burn pruritus is also a common complication that occurs during the healing process. Chung et al. [7] described the pathophysiology of post-burn pruritus and identified the involved mechanisms, cells, and molecules. Based on this, several systematic treatments were proposed, including antihistamines, opioid receptor agonists/antagonists, ondansetron, gabapentin/pregabalin, and antidepressants. Other treatments, such as extracorporeal shockwave therapy, physical treatment (e.g., pressure therapy), or botulinum toxin injections, also appear to have a beneficial impact on post-burn pruritus [7]. Although many potential treatments are available, there is still no consensus on the most effective single treatment for post-burn pruritus. The pathophysiology of post-burn pruritus appears to have both pruritogenic and neuropathic aspects, but the exact mechanisms underlying it still need to be fully understood. Thus, an effective treatment against post-burn pruritus still needs to be developed.

To study the pathophysiological healing processes of burns, reliable burn wound models that incorporate the unique properties of human skin are necessary [8]. Gross-Amat et al. [8] introduced a human ex vivo model for second-degree burns that is maintained under constant tension, resulting in accelerated re-epithelialization through fibroblast activation. This ex vivo model can help investigate the kinetic-based impact on the healing process of burns enabling an adequate evaluation of clinically relevant therapies. Banakh et al. [9] compared commercially available collagen-based and synthetic dermal templates for full-thickness wounds in an in vivo mouse model. After two weeks, the synthetic template demonstrated advantages over the collagen-based template, including significantly higher vascularisation and fibroblast infiltration. While these findings have limited translation, they offer valuable insights regarding selecting dermal templates in a clinical setting and identifying parameters for future studies.

In summary, this Special Issue offers a comprehensive range of studies on the molecular mechanisms associated with burns, wound healing, and scarring. These studies include translational ex-vivo [8] and in-vivo [9] studies, as well as reviews of new treatment modalities [2,3] and pathophysiological aspects of burn-related complications such as scarring [5,6] and pruritus [7]. The current issue provides valuable insights into the current state of the field, which gaps have to be filled, and highlights areas for future research. Therefore, this compilation is highly recommended for anybody interested in the latest advancements in burn management, as it can serve as a guide for future studies.
